# DO MINIMUM STAFFING LEVELS IMPROVE NURSING HOME QUALITY? EVIDENCE FROM CALIFORNIA’S MINIMUM STAFFING REGULATIONS

**DOI:** 10.1093/geroni/igae098.1384

**Published:** 2024-12-31

**Authors:** John Bowblis, Christopher Brunt

**Affiliations:** Miami University, Oxford, Ohio, United States; Georgia Southern University, Statesboro, Georgia, United States

## Abstract

Nursing staff is a key component in providing high quality of care to nursing home (NH) residents, therefore, as of 2021, 39 states have minimum nursing staff regulations. In September of 2023, CMS announced a proposed rule for a first ever federal minimum nursing staff level, even though the effectiveness of these regulations at improving quality is unknown. In this paper, we utilized a difference-in-differences regression framework to understand how changes to minimum staffing regulations impact staffing and quality. In particular, we examine California’s 2018 change to staffing regulations that increased the minimum staffing level from 3.2 to 3.5 hours per resident day (HPRD) and implemented a new 2.4 HPRD minimum requirement for certified nurse aides (CNA). Comparing California to neighboring states that did not change staffing requirements, California NHs increased CNA staffing levels 0.15 HPRD (6.5% increase) and total nursing staff levels of 0.184 HPRD (4.8% increase) on average. Even with these increases, only about 70% of NHs met the new CNA requirement and 90% met the new 3.5 HPRD staffing requirements in 2019. While staffing levels increased, only 3 of the 13 quality measures examined were statistically significant at the 5% or 10% levels. At the 5% level, there was improvement in incontinence among long-stay residents. At the 10% level, there was improvement in pressure ulcers among long-stay residents and the short-stay rehospitalization rate. These results indicate that increasing staffing levels through mandated minimums had small to negligible impact on most aspect of NH quality.

